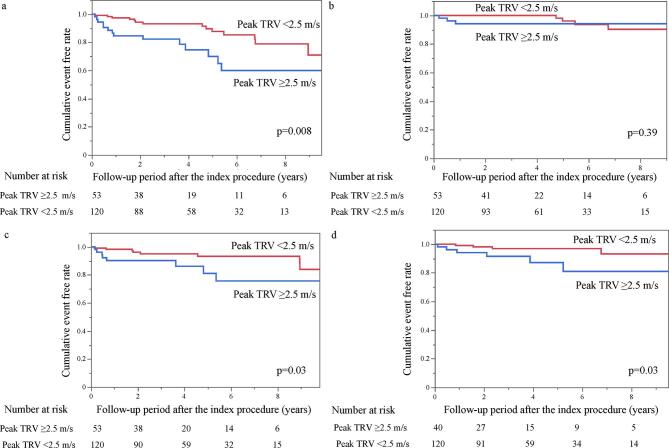# Prognostic significance of diastolic dysfunction in patients with systolic dysfunction undergoing atrial fibrillation ablation

**DOI:** 10.1016/j.ijcha.2022.101079

**Published:** 2022-07-04

**Authors:** Toshiharu Koike, Koichiro Ejima, Shohei Kataoka, Kyoichiro Yazaki, Satoshi Higuchi, Miwa Kanai, Daigo Yagishita, Morio Shoda, Nobuhisa Hagiwara

**Affiliations:** a Department of Cardiology, Tokyo Women’s Medical University, 8-1 Kawada-cho, Shinjuku-ku, Tokyo 162-8666, Japan; b Clinical Research Division for Heart Rhythm Management, Department of Cardiology, Tokyo Women’s Medical University School of Medicine, 8-1 Kawada-cho, Shinjuku-ku, Tokyo 162-8666, Japan

**Keywords:** Atrial fibrillation, Catheter ablation, Diastolic dysfunction, Heart failure, AAD, antiarrhythmic drug, AF, atrial fibrillation, AFMR, atrial functional MR, ATA, atrial tachyarrhythmia, AUC, area under the curve, CRT, cardiac resynchronization therapy, DT, deceleration time, E, early diastolic left ventricular filling velocity, e′, early septal diastolic mitral annular velocity, HF, heart failure, HFH, HF hospitalization, HFrEF, HF with reduced ejection fraction, IQR, interquartile ranges, LA, left atrial, LAVI, LA volume index, LV, left ventricular, LVAD, LV assist device, LVDD, left ventricular diastolic dysfunction, LVEF, LV ejection fraction, LVSD, left ventricular systolic dysfunction, MR, mitral regurgitation, NYHA, New York Heart Association, PAF, paroxysmal AF, PMI, pacemaker implantation, rEF, reduced ejection fraction, ROC, receiver operating characteristic, SD, standard deviations, SHD, structural heart disease, TRV, tricuspid valve regurgitation velocity, VFMR, ventricular functional MR

## Abstract

**Background:**

The relationship between pre-ablation left ventricular diastolic dysfunction (LVDD) and prognosis in patients with left ventricular systolic dysfunction (LVSD) undergoing atrial fibrillation (AF) ablation remains unclear.

**Methods:**

The prognosis of 173 patients with impaired left ventricular ejection fraction (<50%) who underwent AF ablation was examined. The primary outcome was a composite of all-cause mortality, heart failure (HF) hospitalization, and worsening HF symptoms requiring unplanned outpatient intensification of decongestive therapy.

**Results:**

During the follow-up period (median, 3.5 years), the primary outcome after AF ablation occurred in 28 patients (16%). The receiver operating characteristic curve analysis showed that early septal diastolic mitral annular velocity (e′) had a larger area under the curve (0.70) than other LVDD parameters, and optimal cut-off values of LVDD, represented by e′, septal E (early diastolic left ventricular filling velocity)/e′, and peak tricuspid valve regurgitation velocity (TRV), were 5.0 cm/s, 13.2, and 2.5 m/s, respectively. Multivariate analysis revealed that e′ ≤5.0 cm/s (standard hazard ratio [HR], 3.87; 95% confidence interval [CI], 1.73–8.69; p = 0.001), septal E/e′ ≥13.2 (HR, 3.62; 95% CI, 1.60–8.21; p = 0.002), and peak TRV ≥ 2.5 m/s (HR, 2.42; 95% CI, 1.13–5.16; p = 0.02) independently predicted the outcome. Patients with New York Heart Association functional status ≥ III had a 3.3–4.5-fold higher risk of the outcome.

**Conclusions:**

LVDD or severe HF symptoms predict poor outcomes in patients with LVSD undergoing AF ablation. Therefore, patients with LVDD or severe HF symptoms should receive more intensive treatment even after AF ablation.

## Graphical abstract

**Figure:**
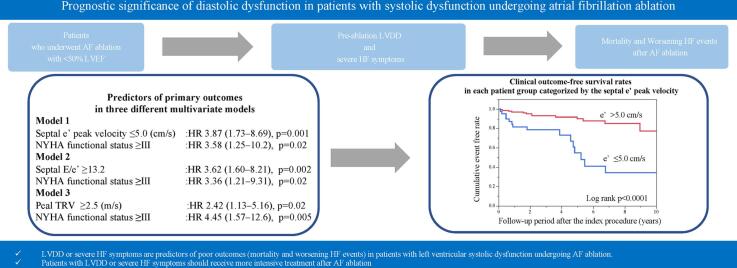

## 1 Introduction

Atrial fibrillation (AF) and heart failure (HF) frequently occur as comorbidities, given that they share the underlying pathophysiology of deteriorating left ventricular (LV) systolic and diastolic function [1], [2]. LV diastolic dysfunction (LVDD) can promote the development of AF [3], [4]. Ablation is a more effective rhythm control strategy for AF than antiarrhythmic drug (AAD) administration [5]. It can even reduce the HF-associated mortality rate in patients with LV systolic dysfunction (LVSD) by improving both LVSD and LVDD [6], [7]. Conversely, AF patients with pre-ablation LVDD have higher recurrence rates of atrial tachyarrhythmia (ATA) after AF ablation than those without pre-ablation LVDD [8], [9]. Regardless of the HF etiology, LVDD in cases of HF with reduced ejection fraction (HFrEF) is associated with higher mortality rates [10]. Nevertheless, the exact relationship between pre-ablation LVDD and poor prognosis, including mortality and HF hospitalization (HFH), in patients with AF and LVSD undergoing AF ablation remains unclear.

Therefore, this study aimed to investigate the relationship between pre-ablation LVDD and prognosis (mortality and worsening HF events) in patients with AF and impaired LV ejection fraction (LVEF) undergoing AF ablation.

## 2 Methods

### 2.1 Study design and participants

**Fig. 1:**
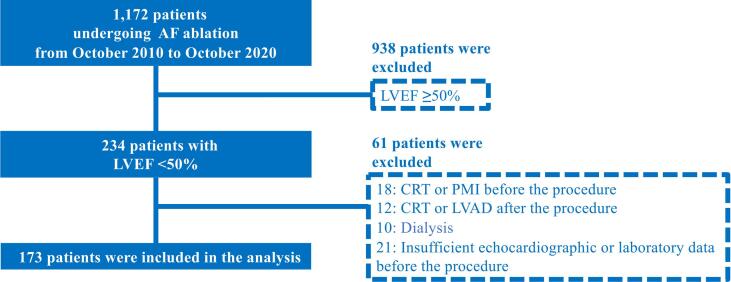
**Flowchart of the selection process.** AF, atrial fibrillation; CRT, cardiac resynchronization therapy; LVAD, left ventricular assist device; LVEF, left ventricular ejection fraction; PMI, pacemaker implantation.

This retrospective observational study screened 1,172 consecutive patients undergoing radiofrequency catheter ablation for AF (Fig. 1). Among those who underwent circumferential pulmonary vein isolation between October 2010 and October 2020, 234 patients with reduced LVEF (<50%) were included in this study. Patients who underwent (1) cardiac resynchronization therapy (CRT), pacemaker implantation (PMI), or LV assist device (LVAD) implantation before the first ablation procedure; (2) CRT or LVAD implantation after the procedure; or (3) hemodialysis before and after the procedure were excluded. Additionally, those whose (4) echocardiography findings were insufficient to define LVSD and LVDD before the procedure and whose (5) laboratory data were insufficient for evaluation were also excluded. The indication for AF ablation was based on previous expert consensus statements [11], [12], [13]. In the month prior to each session, patients underwent multi-dimensional cardiac computed tomography and transthoracic echocardiology. Transesophageal echocardiography was performed in patients with persistent AF and in those with paroxysmal AF (PAF) and a high CHADS_2_ Score for Atrial Fibrillation Stroke Risk (>2 points). This study was conducted in accordance with institutional guidelines as well as the ethical guidelines and principles outlined in the 1975 Declaration of Helsinki and its amendments. The study was approved by the Institutional Review Board of the Tokyo Women’s Medical University (ID: 4190-R). This study is a retrospective study. Moreover, the patients had the choice to opt-out of our hospital’s website. Therefore, patients were not necessarily required to give informed consent to participate in the study.

### 2.2 Catheter ablation

A detailed description of the catheter ablation protocol has been published previously [13]. The ablation catheter comprises a wide circumferential pulmonary vein isolation consisting of a point-by-point radiofrequency application using an image of the three-dimensional (3D) mapping system (CARTO 3; Biosense Webster, Diamond Bar, CA, USA or EnSite velocity™; Abbott, Chicago, IL, USA), two long sheaths, one or two circular multielectrode catheters, and a 3.5 mm open-irrigated tip catheter (ThermoCool, ThermoCool SF, or ThermoCool STSF; Biosense Webster). From February 2013, the empirical superior vena cava isolation technique was performed routinely. The main goal of ablation was the complete isolation of the thoracic veins (the pulmonary veins and the superior vena cava). An adenosine triphosphate infusion was used to confirm the absence of dormant conduction. These procedures were conducted for a minimum of 20 min after isolation of the ipsilateral pulmonary vein pair. If other ATA or non-pulmonary vein foci were triggered, they were targeted for elimination. The AF types (PAF, persistent AF, or long-standing persistent AF) were in accordance with the definitions determined by the 2020 European Society of Cardiology (ESC) guidelines for the diagnosis and management of AF [14]. Here, “non-PAF” refers to persistent or long-standing persistent AF. ATA tachycardia was defined as ATA with a heart rate ≥ 100 bpm [15].

### 2.3 Echocardiographic evaluation

Transthoracic echocardiography was performed by specialists, who were certificated by the Japanese society of echocardiography, in the left lateral decubitus position using the iE 33 (Philips Medical Systems, Eindhoven, The Netherlands), EPIQ 7 (Philips Healthcare), General Electric Vivid E9 (GE Health Medical, Horten, Norway), or Artida (Toshiba Medical Systems, Tokyo, Japan). All the images were stored digitally, and relevant parameters were measured according to the American Society of Echocardiography recommendations [16]. The modified Simpson’s method was used for left atrial (LA) and LV volumetric measurements. Echocardiography was performed before the first ablation procedure. Based on the recent guidelines for HF management [17], structural heart disease (SHD) can be classified into ischemic cardiomyopathy, non-ischemic cardiomyopathy, valvular heart disease, congenital heart disease, hypertrophic cardiomyopathy, or other cardiomyopathies.

According to the 2016 ESC Guidelines for the Diagnosis and Treatment of Acute and Chronic HF [18], reduced ejection fraction (rEF) is defined as an LVEF < 40%. In patients with pre-ablation LVEF data, values are reported as pre-rEF. According to the recent recommendations for evaluation of LVDD in AF patients using echocardiography [19], diastolic function was assessed using the transmitral flow profile from the apical 4-chamber view. The assessment included the early diastolic LV filling velocity (peak E velocity), septal early diastolic mitral annular velocity (septal e′ peak velocity), and deceleration time (DT). The LV filling pressures were estimated by dividing the standard peak E velocity by the septal e′ peak velocity, resulting in the measurement of E/e′ at the septal wall. The estimates were obtained using a color-coded tissue Doppler imaging system with a four-chamber view. Besides the transmitral flow profile, the peak tricuspid valve regurgitation velocity (TRV) was assessed to evaluate the diastolic function from multiple views by searching for the best envelope and maximal velocity [19]. The peak TRV was measured from the spectral profile of the tricuspid jet. The highest *trans*-valvular velocity was used for calculation of the peak TRV. The severity of the mitral regurgitation (MR) was determined using a multiparametric approach, which included an assessment of the effective regurgitant orifice area using the proximal isovelocity surface area method or the MR volume using the Doppler volumetric method. Primary MR, ventricular functional MR (VFMR), and atrial functional MR (AFMR) were classified according to the 2021 ESC/EACTS guidelines for the management of valvular heart disease [20].

### 2.4 Follow-up

Unless ATA recurrence occurred, AADs were not used after the first ablation procedure. Patients visited the outpatient clinic at 1, 3, 6, 9, and 12 months after the procedure and every 6 months thereafter. Their medical records were reviewed, and patient admission or death during the follow-up period was noted. ATA recurrence was assessed using 24-h ambulatory electrocardiographic monitoring every 3 months in the first year and every 6 months thereafter. A portable electrocardiogram (HCG-801R; Omron, Kyoto, Japan) was recommended for patients who did not have documented electrocardiograms but had frequent symptoms, whereas pulse checks two to three times/day were recommended for asymptomatic patients. ATA recurrence was defined as symptomatic and/or documented ATA detected via 12-lead electrocardiography, 24-h ambulatory electrocardiographic monitoring, or portable electrocardiography after a 3-month blanking period, regardless of the use of AADs.

The primary outcome was a composite of all-cause mortality, HFH, and worsening HF symptoms requiring unplanned outpatient intensification of decongestive therapy, whichever occurred first. With reference to previous studies, worsening HF symptoms requiring unplanned outpatient intensification of decongestive therapy were defined as (1) new or progressive HF symptoms, including substantial weight gain, worsening dyspnea, newly elevated jugular venous pressure, development of pulmonary rales, hepatic congestion, cool extremities, or lower-extremity edema [21]; and (2) unplanned intensification of oral or intravenous decongestive therapy with loop diuretics or addition of a thiazide diuretic to loop diuretics (except for guideline-directed medical therapy) without formal inpatient hospitalization necessitating an overnight stay [21], [22], [23], [24]. The secondary outcomes were all-cause mortality, HFH, and worsening HF symptoms requiring unplanned outpatient intensification of decongestive therapy, separately.

### 2.5 Statistical methods

According to the distribution and variance of continuous variables, the variables are presented as means and standard deviations (SD) or as medians with interquartile ranges (IQR). The Student *t*-test and the Wilcoxon test were used to compare continuous variables between the groups with and without the primary outcome. Categorical variables are presented as numbers and percentages and were compared using the chi-squared test or Fisher exact test. In the receiver operating characteristic (ROC) curve analysis, the area under the curve (AUC) and the optimal cut-off value for echocardiographic parameters of diastolic function were evaluated to predict the primary outcome after the first ablation procedure. The incidence of the clinical outcome was assessed using the Kaplan–Meier method, and the significance of differences among groups was derived using the log-rank test. The Cox proportional hazards model was used to evaluate the predictors of the primary outcome in univariate and multivariate analyses. According to the results of the univariate analysis, multivariate analysis was performed using three models that included relevant covariates. All analyses were performed using JMP software version 15 (SAS Institute, Cary, NC, USA). A two-sided p-value of < 0.05 was considered statistically significant.

## 3 Results

Out of 234 patients with impaired LVEF (<50%), 18 underwent CRT or PMI before the first ablation procedure, 12 underwent CRT after the first ablation procedure, 10 underwent dialysis before and after the procedure, and 21 did not have sufficient echocardiographic or laboratory data before the procedure. These 61 patients were excluded from the study, and the remaining 173 patients (average age, 61, SD = 11 years; sex, 84% and 16% male and female participants; patients with known SHD, 39%) were selected for further evaluation. The mean pre-ablation LVEF was 43%. Overall, 109, 56, and seven patients required one, two, and three AF ablation procedures, respectively. During the follow-up period after the first ablation procedure, atrial tachycardia, atrial flutter, and AF occurred in 18 (10%), six (3%), and 67 (39%) patients, respectively.

Table 1 summarizes the characteristics of patients with and without the primary outcome at baseline. The median follow-up period after the first ablation procedure was 3.5 (interquartile range, 2.1–6.5) years. During the follow-up period, the primary outcome occurred in 28 (16%) patients. There were six (4%) cases of all-cause mortality (cardiac issues, n = 2; any malignancies, n = 2; and other issues, n = 2), 13 (8%) cases of HFH, and nine cases (5%) of worsening HF symptoms requiring unplanned outpatient intensification of decongestive therapy. ATA recurrence was more frequent in patients with the primary outcome than those without the primary outcome. Known SHD was more frequent in patients with the outcome than in those without the outcome. Moreover, the New York Heart Association (NYHA) functional status was more severe in patients with the outcome than in those without the outcome. LVEF at baseline was higher in patients without the outcome than in those with the outcome. LVDD (represented by septal e′ peak velocity, septal E/e′, and peak TRV) was more severe in patients with the outcome than in those without the outcome. With regards to the MR morphology, no patient had primary MR or AFMR, and 10 patients had VFMR before AF ablation. After AF ablation, MR improved to less than moderate MR in seven (70%) out of 10 patients who had moderate or severe VFMR before AF ablation. Standard medications for HF stabilization were more commonly prescribed in patients with the outcome than in those without the outcome.

**Table 1:** Baseline characteristics of the total study population and patients with and without the composite of all-cause mortality, HFH, and worsening HF symptoms requiring unplanned outpatient intensification of decongestive therapy.

	All (n = 173)	With the primary outcome (n = 28)	Without the primary outcome (n = 145)	*p* value
Age (years)	61 ± 11	63 ± 13	61 ± 10	0.33
Male sex	145 (84)	22 (79)	123 (84)	0.41
BMI (kg/m^2^)	24 ± 3	24 ± 3	24 ± 3	0.74
Non-PAF	107 (62)	14 (50)	93 (64)	0.16
History of AF (months)	60 [23–120]	11 [3–69]	24 [6–60]	0.29
ATA recurrence	85 (49)	19 (68)	66 (46)	0.03
Total sessions	1 [1], [2]	2 [1], [2]	1 [1], [2]	0.05
Hypertension	85 (49)	12 (43)	73 (50)	0.47
Diabetes	27 (16)	5 (18)	22 (15)	0.72
Stroke	16 (9)	5 (18)	11 (8)	0.09
Vascular disease	12 (7)	3 (11)	9 (6)	0.39
CHADS_2_ score	1 [1], [2]	2 [1], [2], [3], [4]	1 [1], [2]	0.02
NYHA functional status				<0.0001
Ⅰ	80 (46)	3 (11)	77 (53)	
Ⅱ	83 (48)	20 (71)	63 (43)	
III	8 (5)	5 (18)	3 (2)	
Ⅳ	2 (1)	0 (0)	2 (1)	
Known SHD	68 (39)	18 (64)	50 (34)	0.003
Non-ischemic cardiomyopathy	32 (19)	9 (32)	23 (16)	
Ischemic cardiomyopathy	22 (13)	6 (21)	16 (11)	
Valvular heart disease	10 (6)	2 (7)	8 (6)	
Congenital heart disease	8 (5)	2 (7)	6 (4)	
Hypertrophic cardiomyopathy	8 (5)	3 (11)	5 (3)	
Creatinine (mg/dL)	0.94 [0.83–1.12]	1.00 [0.86–1.20]	0.94 [0.83–1.11]	0.21
eGFR (mL/min/1.73 m^2^)	61 ± 16	55 ± 17	62 ± 15	0.03
CKD (eGFR < 60 mL/min/1.73 m^2^)	88 (51)	18 (64)	70 (48)	0.12
Medication at baseline				
Warfarin	80 (46)	18 (64)	62 (43)	0.04
DOAC	93 (54)	10 (36)	83 (57)	0.04
β blocker	120 (69)	23 (82)	97 (67)	0.11
ACE-I/ARB	100 (58)	23 (82)	77 (53)	0.004
ARNI	0 (0)	0 (0)	0 (0)	0
MCR antagonist	46 (27)	15 (54)	31 (21)	0.0004
SGLT-2 inhibitor	1 (1)	0 (0)	1 (1)	0.66
Pre-AAD	82 (47)	17 (61)	65 (45)	0.12
Echocardiographic parameters				
HR during echocardiography	77 [62–92]	70 [61–89]	79 [63–94]	0.27
ATA tachycardia during echocardiography	25 (14)	5 (18)	20 (14)	0.58
LVEF (%)	43 [35–48]	40 [31–46]	44 [37–48]	0.02
LVEDV (mL)	142 [116–167]	138 [120–185]	143 [115–166]	0.33
LVESV (mL)	79 [63–102]	81 [67–129]	79 [62–98]	0.23
LVEDVI (mL/m^2^)	79 [54–100]	84 [69–139]	79 [66–107]	0.10
LVESVI (mL/m^2^)	45 [35–57]	50 [38–69]	44 [35–55]	0.07
Pre-rEF	62 (35)	14 (50)	47 (32)	0.07
LAV (mL)	81 ± 26	90 ± 28	79 ± 25	0.03
LAVI (mL/m^2^)	46 ± 15	52 ± 17	45 ± 14	0.03
Peak E-wave velocity (cm/s)	75 [60–88]	65 [55–98]	76 [61–87]	0.37
Septal e′ peak velocity (cm/s)	7.2 [5.2–8.7]	4.9 [3.7–6.5]	7.4 [5.4–9.0]	0.0008
Septal E/e′	10.3 [8.1–14.2]	13.8 [9.37–20.2]	10.1 [8.05–12.9]	0.02
Peak TRV (m/s)	2.2 [2.0–2.5]	2.5 [2.1–2.8]	2.2 [2.0–2.5]	0.02
DT (ms)	162 [127–204]	167 [124–232]	161 [129–201]	0.41
MR ≥ moderate	10 (6)	2 (7)	8 (6)	0.74
Primary MR ≥ moderate	0 (0)	0 (0)	0 (0)	0
Atrial functional MR ≥ moderate	0 (0)	0 (0)	0 (0)	0
Ventricular functional MR ≥ moderate	10 (0)	2 (7)	8 (6)	0.74

Data are presented as mean ± SD, n (%), or median [interquartile range].

Xxxx ACEI, angiotensin-converting enzyme inhibitor; AF, atrial fibrillation; ARB, angiotensin receptor blocker; ARNI, angiotensin receptor neprilysin inhibitor; ATA, atrial tachyarrhythmia; ATA tachycardia, atrial tachyarrhythmia with heart rate ≥ 100 bpm; BMI, body mass index; CKD, chronic kidney disease; DOAC, direct oral anticoagulant; DT, deceleration time; E, early diastolic left ventricular filling velocity; eGFR, estimated glomerular filtration rate; HFH, heart failure hospitalization; HR, hear rate; LAV, left atrial volume; LAVI, left atrial volume index; LVEDV, left ventricular end-diastolic volume; LVEDVI, left ventricular end-diastolic volume index; LVEF, left ventricular ejection fraction; LVESV, left ventricular end-systolic volume; LVESVI, left ventricular end-systolic volume index; MCR, mineralocorticoid receptor; MR, mitral valve regurgitation; Non-PAF, non-paroxysmal atrial fibrillation (meaning persistent atrial fibrillation or long-standing persistent atrial fibrillation); NYHA, New York Heart Association; PAF, paroxysmal atrial fibrillation; peak TRV, peak tricuspid valve regurgitation velocity; Pre-AAD, oral administration of antiarrhythmic drug before the procedure; Pre-rEF, Pre-ablation reduced ejection fraction (left ventricular ejection fraction < 40%); Septal e′ peak velocity, septal early diastolic mitral annular velocity; SGLT-2, sodium glucose cotransporter 2; SHD, structural heart disease.

The AUC values in the ROC curve analysis for predicting the primary outcome in AF patients are presented in Table 2. The septal e′ peak velocity demonstrated a higher AUC (0.70) than other parameters for diastolic function. The ROC curve analysis demonstrated that the optimal cut-off value of the septal e′ peak velocity was 5.0 cm/s. Applying the septal e′ peak velocity cut-off value ≤ 5.0 cm/s to the primary outcome in all patients resulted in 57 true positives, 83 true negatives, 17 false positives, and 43 false negatives. The sensitivity, specificity, positive predictive value, and negative predictive values of septal e′ peak velocity were 57%, 83%, 40%, and 91%, respectively.

**Table 2:** Area under the curve for left ventricular diastolic dysfunction at baseline.

Parameters	Area under the curve	Cut-off value
Septal e′ peak velocity (cm/s)	0.70	5.0
Septal E/e′	0.65	13.2
Peak TRV (m/s)	0.64	2.5
DT (ms)	0.55	194

Xxx DT, deceleration time; E, early diastolic left ventricular filling velocity; Peak TRV, peak tricuspid valve regurgitation velocity; Septal e′ peak velocity, septal early diastolic mitral annular velocity.

Table 3 shows the hazard ratios (HRs) of the primary outcome based on the univariate analysis. NYHA functional status ≥ III, known SHD, pre-rEF state, septal e′ peak velocity cut-off value ≤ 5.0 cm/s, septal E/e′ cut-off value ≥ 13.2, and peak TRV cut-off value ≥ 2.5 m/s were significantly associated with the primary outcome. On the contrary, ATA recurrence, non-PAF, and the morphology of severe or moderate MR were not significantly associated with the primary outcome. According to the results of the univariate analysis, multivariate analysis was performed using three different models that included variables that were considered to be related to the primary outcome. Each model included NYHA functional status ≥ III, known SHD, and pre-rEF (Table 4). Regarding LVDD, Models 1, 2, and 3 included septal e′ peak velocity cut-off value ≤ 5.0 cm/s, septal E/e′ cut-off value ≥ 13.2, and peak TRV cut-off value ≥ 2.5 m/s, respectively. In each assessment, patients with NYHA functional status ≥ III had a 3.3–4.5-fold higher risk of the outcome than those without this status. Regarding LVDD, the septal e′ peak velocity cut-off value ≤ 5.0 cm/s (HR, 3.87; 95% confidence interval [CI], 1.73–8.69; p = 0.001), septal E/e′ cut-off value ≥ 13.2 (HR, 3.62; 95% CI, 1.60–8.21; p = 0.002), and peak TRV cut-off value ≥ 2.5 m/s (HR, 2.42; 95% CI, 1.13–5.16; p = 0.02) independently predicted the outcome. Supplementary table 1 shows the baseline characteristics of the total patients with and without pre-ablation LVDD, which include a septal e′ peak velocity cut-off value ≤ 5.0 cm/s, septal E/e′ cut-off value ≥ 13.2, or peak TRV cut-off value ≥ 2.5 m/s.

**Table 3:** Predictors of the primary outcome in the univariate analysis using Cox regression analysis.

Variables	HR (95% CI)	*p* value
Age (years)	1.02 (0.99–1.07)	0.14
Male	0.67 (0.27–1.67)	0.39
BMI	0.99 (0.88–1.12)	0.93
Non-PAF	0.71 (0.34–1.50)	0.37
History of AF (months)	0.61 (0.03–5.91)	0.71
ATA recurrence	1.72 (0.78–3.84)	0.18
Hypertension	0.85 (0.40–1.81)	0.68
Diabetes	1.63 (0.61–4.31)	0.33
Vascular disease	2.20 (0.66–7.33)	0.20
NYHA functional status ≥ III	5.94 (2.19–16.1)	0.0005
Known SHD	3.45 (1.59–7.49)	0.002
Baseline CKD	2.10 (0.97–4.56)	0.06
HR during echocardiography (bpm)	0.49 (0.06–3.16)	0.48
ATA tachycardia during echocardiography	1.53 (0.58–4.07)	0.39
Pre-rEF	2.38 (1.13–5.02)	0.02
LAVI ≥ 34 (mL/m^2^)	2.12 (0.74–6.15)	0.16
Septal e′ peak velocity ≤ 5.0 (cm/s)	5.04 (2.39–10.7)	<0.0001
Septal E/e′ ≥13.2	4.65 (2.18–9.92)	<0.0001
Peak TRV ≥ 2.5 (m/s)	2.63 (1.25–5.52)	0.01
DT ≤ 194 (ms)	0.71 (0.34–1.51)	0.37
Ventricular functional MR ≥ moderate	1.93 (0.45–8.22)	0.37
Warfarin	1.16 (0.51–2.63)	0.72
Pre-AAD	1.27 (0.59–2.72)	0.55

Xxx AAD, antiarrhythmic drug; AF, atrial fibrillation; ATA tachycardia, atrial tachyarrhythmia with heart rate ≥ 100 bpm; ATA, atrial tachyarrhythmia; BMI, body mass index; CI, confidence interval; CKD, chronic kidney disease; DT, deceleration time; E, early diastolic left ventricular filling velocity; HR, hazard ratio; HR, heart rate; LAVI, left atrial volume index; MR, mitral regurgitation; Non-PAF, non-paroxysmal atrial fibrillation (meaning persistent atrial fibrillation or long-standing persistent atrial fibrillation); NYHA, New York Heart Association; Peak TRV, peak tricuspid valve regurgitation velocity; Pre-AAD, oral administration of antiarrhythmic drug before the procedure; Pre-rEF, Pre-ablation reduced ejection fraction (left ventricular ejection fraction < 40%); Septal e′ peak velocity, septal early diastolic mitral annular velocity; SHD, structural heart disease.

**Table 4:** Predictors of the primary outcome in three different models in the multivariate analysis using Cox regression analysis.

Variables	HR (95% CI)	p value
**Model 1**		
NYHA functional status **≥** III	3.58 (1.25–10.2)	0.02
Known SHD	1.78 (0.77–4.16)	0.18
Pre-rEF	1.74 (0.78–3.89)	0.17
Septal e′ peak velocity ≤ 5.0 (cm/s)	3.87 (1.73–8.69)	0.001
**Model 2**		
NYHA functional status **≥** III	3.36 (1.21–9.31)	0.02
Known SHD	1.80 (0.75–4.33)	0.19
Pre-rEF	1.78 (0.81–3.93)	0.15
Septal E/e′ ≥13.2	3.62 (1.60–8.21)	0.002
**Model 3**		
NYHA functional status **≥** III	4.45 (1.57–12.6)	0.005
Known SHD	2.48 (1.10–5.60)	0.03
Pre-rEF	1.47 (0.67–3.20)	0.34
Peak TRV ≥ 2.5 (m/s)	2.42 (1.13–5.16)	0.02

Xxx CI, confidence interval; E, early diastolic left ventricular filling velocity; HR, hazard ratio. NYHA, New York Heart Association; Peak TRV, peak tricuspid valve regurgitation velocity; Pre-rEF, Pre-ablation reduced ejection fraction (left ventricular ejection fraction < 40%); septal e′ peak velocity, septal early diastolic mitral annular velocity; SHD, structural heart disease.

**Fig. 2:**
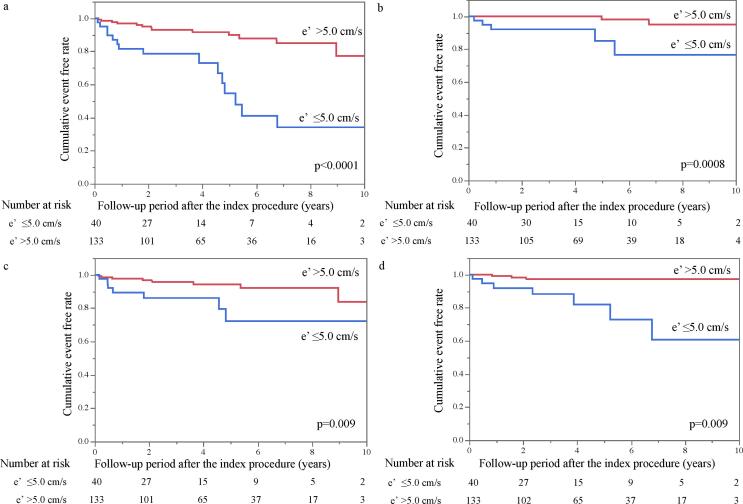
**Clinical outcome-free survival rates in each patient group categorized by septal e′ peak velocity.** Kaplan–Meier curves showing the difference in the cumulative rate of (a) the composite of (b) all-cause mortality, (c) heart failure hospitalization, and (d) worsening heart failure symptoms requiring unplanned outpatient intensification of decongestive therapy between the septal e′ peak velocity ≤ 5.0 cm/s group and the septal e′ peak velocity > 5.0 cm/s group. e′, septal early diastolic mitral annular velocity.

**Supplementary figure 1:**
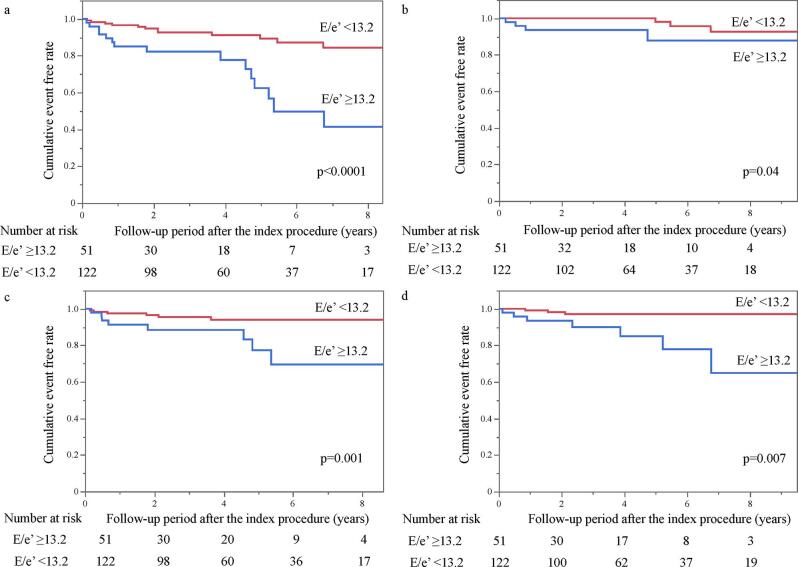


A significant difference in the incidence of the primary outcome was identified between patients with septal e′ peak velocity ≤ 5.0 cm/s and those with septal e′ peak velocity > 5.0 (p < 0.0001; Fig. 2). The cumulative ratios of the primary outcome in patients with septal e′ peak velocity ≤ 5.0 cm/s and septal e′ peak velocity > 5.0 cm/s were 21% and 7%, respectively, at 3 years after the first ablation procedure and 45% and 10%, respectively, at 5 years after the procedure (Fig. 2a). Similar findings were noted between the two groups with respect to the incidence of all-cause mortality (p = 0.0008; Fig. 2b), HFH (p = 0.009; Fig. 2c), and worsening HF symptoms requiring unplanned outpatient intensification of decongestive therapy (p = 0.009; Fig. 2d). Similar findings regarding the cumulative ratios of the primary and secondary outcomes were demonstrated between the two groups categorized by the remaining two parameters of LVDD—namely, septal E/e′ cut-off value ≥ 13.2 and peak TRV cut-off value ≥ 2.5 m/s (Supplemental Figs. 1 and 2).

## 4 Discussion

In this study, we explored the relationship between pre-ablation LVDD and prognosis in patients with AF and LVSD undergoing AF ablation. First, the echocardiographic parameter for diastolic function, represented by the septal e′ peak velocity, had a moderate predictive value for the primary outcome using the AUC values in the ROC curve analysis. Second, on multivariate analysis using three different models (adjusted for confounders other than LVDD), the cut-off value of the three LVDD parameters—septal e′ peak velocity, septal E/e,' and peak TRV—emerged as independent predictors of the outcomes. Third, NYHA functional status ≥ III was an independent predictor of the primary outcome. Finally, patients with LVDD, which is defined as septal e′ peak velocity ≤ 5.0 cm/s or septal E/e′ ≥13.2 or peak TRV ≥ 2.5 m/s, had a poorer prognosis for the primary and secondary outcomes than those without LVDD.

Some previous studies found that 10–40% of patients with AF treated by AF ablation have concomitant LVDD [8], [9]. Conversely, other studies have shown that the presence and severity of LVDD are independent predictors of the development of AF [3], [25]. Thus, AF and LVDD often coexist and interact with each other. In a previous study not limited to patients with AF, Benfari et al. showed that 53% of patients with HFrEF had concomitant LVDD, which was represented by an E/e′ value > 14 [10]. Herein, 51% of the included patients had LVDD, which was defined as a septal E/e′ value ≥ 13.2, septal e′ peak velocity ≤ 5.0 cm/s, or peak TRV ≥ 2.5 m/s. The proportion of patients with LVDD in the present study was similar to that reported in a previous study [10].

In patients with AF, the Doppler assessment of LV diastolic function is limited by the irregular rhythm and the loss of organized atrial activity [19]. In general, when LVEF is impaired in patients with AF, mitral DT (≤160 ms) has reasonable accuracy in predicting the increased LV filling pressure [26], [27]. Other Doppler measurements that could be applied include e′ peak velocity and E/e′ [19], [26], [27], [28], [29], [30], [31]. Okada et al. demonstrated that septal e′ peak velocity effectively enhanced myocardial relaxation and concluded that septal e′ peak velocity was more valuable in LVDD assessment than lateral e′ [28]. In patients with AF, e′ peak velocity correlated well with tau, which represents myocardial relaxation ability [29]. Moreover, peak TRV ≥ 2.8 m/s is suggestive of elevated LA pressure in patients with AF [19]. Therefore, in the present study, DT, e′ peak velocity, E/e′, and peak TRV were used in LVDD assessment. However, the cut-off values of these LV diastolic function parameters were not determined for predicting prognosis (mortality and worsening HF events) in patients with AF and impaired LVEF undergoing AF ablation. Therefore, in the present study, an ROC curve analysis was performed to determine the cut-off value of LV diastolic function for predicting prognosis individually.

Several previous studies have reported that the accuracy of LVDD, represented by E/e′ and e′ peak velocity, was low in patients after CRT and right ventricular pacing [19], [32], [33]. Therefore, in the present study, patients who underwent CRT or PMI before the first ablation procedure were excluded. Hence, the present study more accurately evaluated the relationship between LVDD (represented by a lower e′ peak velocity and higher E/e′) and poor prognosis than earlier studies [8], [9].

AF and HF can interact through atrial cardiomyopathy, which is any complex of structural or electrophysiological changes affecting the atria with the potential to produce clinically relevant manifestations. Especially, HF can lead to atrial cardiomyopathy, which can further initiate and perpetuate AF [34]. Moreover, AF can lead to atrial cardiomyopathy and HF [17], [34]. Conversely, AF ablation can reduce HF-associated mortality in patients with HF and impaired LVEF [5], [6]. Moreover, early rhythm control therapy using a combination of AADs and AF ablation can improve the prognosis in patients with AF and HF [35]. Successful AF ablation can improve LV systolic and diastolic function [2], [7], [36]. Conversely, some studies have demonstrated that pre-ablation LVDD could be associated with ATA recurrence after AF ablation [8], [9]. Nevertheless, to the best of our knowledge, no study reported that pre-ablation LVDD was associated with poor prognosis, including mortality and worsening HF events, after AF ablation.

Notably, our results revealed that pre-ablation LVDD (defined as septal e′ peak velocity ≤ 5.0 cm/s, septal E/e′ ≥13.2, peak TRV ≥ 2.5 m/s) was an independent risk factor for poor prognosis in patients with AF and LVSD undergoing AF ablation. In a previous study, lower e′ peak velocity independently predicted cardiovascular mortality [37]. Benfari et al. showed that higher E/e′ values using e′ peak velocity were associated with increased mortality rates in patients with HFrEF regardless of the underlying etiology [10]. In patients with AF, peak TRV > 2.8 m/s may be useful as a surrogate marker of elevated LV filling pressure [19]. Additionally, a previous study has shown that elevated tricuspid valve regurgitation pressure gradient using peak TRV was associated with adverse cardiovascular event and mortality in patients with AF [38]. These previous findings regarding the relationship between LVDD and poor prognosis support our results.

In the present study, LAVI ≥ 34 mL/m^2^, which is a standard cuoff for left atrial enlargement (19), was not significantly associated with poor prognosis. LVDD can prompt atrial dysfunction including atrial enlargement as a part of atrial cardiomyopathy, which can contribute to poor prognosis (19,34). Therefore, although left atrial enlargement was not associated with poor prognosis, atrial dysfunction other than left atrial enlargement due to LVDD may have resulted in LVDD being a poor prognosis factor in the present study.

Recently, we reported that pre-ablation LVDD in AF patients with LVSD undergoing AF ablation, defined as low septal e′ peak velocity, could predict non-improvement of LVEF after AF ablation [39]. Moreover, we reported that persistent rEF after AF ablation was associated with poor outcomes [40]. Findings of the present and previous studies suggest that pre-ablation LVDD with LVSD is associated with non-improvement of LVEF after AF ablation, which may lead to a poor prognosis. Although previous studies have shown that AF ablation in patients with LVSD is associated with better prognosis than medical therapy [6], [41], [42], those with both pre-ablation LVDD and LVSD should be observed more carefully after AF ablation than those without LVDD. In such patients, intensification of standard medications for HF stabilization or indications for cardiac implantable electronic devices should be considered to improve prognosis.

Overall, in the present study, 10 patients (6%) had HF symptoms with NYHA functional status ≥ III. Moreover, NYHA functional status ≥ III independently predicted poor prognosis in this study. In the CASTLE AF trial, which compared the prognosis of AF patients with LVEF < 35% treated by AF ablation with that of patients who received drug treatment, patients with NYHA functional status ≥ III were less likely to benefit from AF ablation than those with NYHA functional status of II. [6], [42] These previous studies supported our findings [6], [41].

### 4.1 Limitations

The present study had some limitations. First, this study was a retrospective study based on patients attending one medical center/facility, which may have led to selection bias. Second, we analyzed a small number of patients and events. Third, we only analyzed patients undergoing AF ablation and could not therefore evaluate the discrete relationship between LVDD and prognosis in patients with LVSD and AF who did not undergo AF ablation. Fourth, patients who had undergone CRT or PMI before the procedure were excluded; hence, we could not evaluate the relationship between prognosis and diastolic function in patients with AF undergoing CRT or PMI before AF ablation. Finally, we were unable to evaluate the relationship between prognosis and other parameters for diastolic function than those assessed in this study and prognosis.

### 4.2 Conclusions

During a relatively protracted follow-up period, pre-ablation LVDD (represented by a lower septal e′ peak velocity, higher septal E/e′, and higher peak TRV) and a more severe NYHA functional status were independently associated with poor prognosis in AF patients with impaired LVEF undergoing AF ablation. In cases with impaired LVEF, patients with pre-ablation LVDD or severe HF symptoms should be carefully monitored even after AF ablation.

#### CRediT authorship contribution statement

**Toshiharu Koike:** Conceptualization, Data curation, Investigation, Formal analysis, Writing – original draft. **Koichiro Ejima:** Writing – review & editing. **Shohei Kataoka:** Data curation. **Kyoichiro Yazaki:** Data curation. **Satoshi Higuchi:** Data curation. **Miwa Kanai:** Data curation. **Daigo Yagishita:** Data curation. **Morio Shoda:** Writing – review & editing. **Nobuhisa Hagiwara:** Writing – review & editing.

## Declaration of Competing Interest

The authors declare that they have no known competing financial interests or personal relationships that could have appeared to influence the work reported in this paper.

## Figures and Tables

**Supplementary figure 2:**