# Supplementary material for: Prognostic significance of diastolic dysfunction in patients with systolic dysfunction undergoing atrial fibrillation ablation
Source: Int J Cardiol Heart Vasc. 2022 Jul 4;41:101079. doi: 10.1016/j.ijcha.2022.101079 (PMC9260613; doi:10.1016/j.ijcha.2022.101079)
Supplement: Supplementary data 1 [file mmc1.docx]

**Supplementary Table 1. Baseline characteristics of the total patients with and without pre-ablation LVDD**

|  | **All (n=173)** | **With LVDD (n=88)** | **Without LVDD**  **(n=85)** | ***p* value** |
| --- | --- | --- | --- | --- |
| Age (years) | 61±11 | 65±10 | 57±10 | <0.0001 |
| Male sex | 145 (84) | 71 (81) | 74 (87) | 0.25 |
| BMI (kg/m^2^) | 24 [22–26] | 23 [21–26] | 24 [22–27] | 0.05 |
| Non-PAF | 107 (62) | 56 (64) | 51 (60) | 0.62 |
| History of AF (months) | 60 [23–120] | 16 [4–69] | 24 [9–60] | 0.09 |
| ATA recurrence | 85 (49) | 42 (48) | 43 (51) | 0.71 |
| Total sessions | 1 [1–2] | 1 [1–2] | 1 [1–2] | 0.05 |
| Hypertension | 85 (49) | 45 (51) | 40 (47) | 0.59 |
| Diabetes | 27 (16) | 19 (22) | 8 (9) | 0.03 |
| Stroke | 16 (9) | 9 (10) | 7 (8) | 0.65 |
| Vascular disease | 12 (7) | 9 (10) | 3 (4) | 0.08 |
| CHADS_2_ score | 1 [1–2] | 2 [1–3] | 1 [0–1] | 0.0003 |
| NYHA functional status |  |  |  | 0.01 |
| Ⅰ | 80 (46) | 32 (36) | 48 (56) |  |
| Ⅱ | 83 (48) | 50 (57) | 33 (39) |  |
| Ⅲ | 8 (5) | 6 (7) | 2 (2) |  |
| Ⅳ | 2 (1) | 0 (0) | 2 (2) |  |
| Known SHD | 68 (39) | 46 (52) | 22 (26) | 0.0004 |
| Non-ischemic cardiomyopathy | 32 (19) | 18 (20) | 14 (16) |  |
| Ischemic cardiomyopathy | 22 (13) | 16 (18) | 6 (7) |  |
| Valvular heart disease | 10 (6) | 9 (10) | 1 (1) |  |
| Congenital heart disease | 8 (5) | 5 (6) | 3 (4) |  |
| Hypertrophic cardiomyopathy | 8 (5) | 8 (9) | 0 (0) |  |
| Creatinine (mg/dL) | 0.94 [0.83–1.12] | 0.97 [0.82–1.18] | 0.94 [0.85–1.08] | 0.46 |
| eGFR (mL/min/1.73 m^2^) | 61±16 | 58±18 | 64±13 | 0.01 |
| CKD (eGFR <60 mL/min/1.73 m^2^) | 88 (51) | 50 (57) | 38 (45) | 0.11 |
| Medication at baseline |  |  |  |  |
| Warfarin | 80 (46) | 39 (44) | 41 (48) | 0.61 |
| DOAC | 93 (54) | 49 (56) | 44 (52) | 0.61 |
| β blocker | 120 (69) | 62 (70) | 58 (68) | 0.75 |
| ACE-I/ARB | 100 (58) | 57 (65) | 43 (51) | 0.06 |
| ARNI | 0 (0) | 0 (0) | 0 (0) | 0 |
| MCR antagonist | 46 (27) | 26 (30) | 20 (24) | 0.37 |
| SGLT-2 inhibitor | 1 (1) | 0 (0) | 1 (1) | 0.31 |
| Pre-AAD | 82 (47) | 39 (44) | 43 (51) | 0.41 |
| Echocardiographic parameters |  |  |  |  |
| HR during echocardiography | 77 [62–92] | 77 [60–94] | 79 [67–91] | 0.61 |
| ATA tachycardia during echocardiography | 25 (14) | 14 (16) | 11 (13) | 0.58 |
| LVEF (%) | 43 [35–48] | 43 [33­–47] | 44 [38–48] | 0.09 |
| LVEDV (mL) | 142 [116–167] | 139 [117–167] | 139 [117–172] | 0.73 |
| LVESV (mL) | 79 [63–102] | 82 [64–109] | 78 [62–97] | 0.39 |
| LVEDVI (mL/m^2^) | 79 [54–100] | 82 [68–98] | 77 [67–89] | 0.10 |
| LVESVI (mL/m^2^) | 45 [35–57] | 47 [37–61] | 42 [35–51] | 0.09 |
| Pre-rEF | 61 (35) | 35 (40) | 26 (31) | 0.21 |
| LAV (mL) | 81±26 | 86±27 | 75±23 | 0.008 |
| LAVI (mL/m^2^) | 46±15 | 42±13 | 50±16 | 0.0008 |
| Peak E-wave velocity (cm/s) | 75±21 | 82 [61–98] | 72 [59–81] | 0.02 |
| Septal e′ peak velocity (cm/s) | 7.2 [5.2–8.7] | 5.3 [4.4–7.5] | 8.4 [7.1–9.7] | <0.0001 |
| Septal E/e′ | 10.3 [8.1–14.2] | 14.2 [10.5–18.8] | 9.0 [7.5–10.3] | <0.0001 |
| Peak TRV (m/s) | 2.2 [2.0–2.5] | 2.5 [2.2–2.7] | 2.0 [1.9–2.2] | <0.0001 |
| DT (ms) | 162 [127–204] | 165 [123–211] | 161 [137–197] | 0.77 |
| MR ≥moderate | 10 (6) | 9 (10) | 1 (1) | 0.01 |
| Primary MR ≥moderate | 0 (0) | 0 (0) | 0 (0) | 0 |
| Atrial functional MR ≥moderate | 0 (0) | 0 (0) | 0 (0) | 0 |
| Ventricular functional MR ≥moderate | 10 (0) | 9 (10) | 1 (1) | 0.01 |

Data are presented as mean ± SD, n (%), or median [interquartile range]. Herein, LVDD was defined as the presence of a septal e’ peak velocity ≤5.0 cm/s or septal E /e′ ≥13.2 or peak TRV ≥2.5 m/s.

ACEI, angiotensin-converting enzyme inhibitor; AF, atrial fibrillation; ARB, angiotensin receptor blocker; ARNI, angiotensin receptor neprilysin inhibitor; ATA, atrial tachyarrhythmia; ATA tachycardia, atrial tachyarrhythmia with heart rate ≥100 bpm; BMI, body mass index; CKD, chronic kidney disease; DOAC, direct oral anticoagulant; DT, deceleration time; E, early diastolic left ventricular filling velocity; eGFR, estimated glomerular filtration rate; HFH, heart failure hospitalization; HR, hear rate; LAV, left atrial volume; LAVI, left atrial volume index; LVDD, left ventricular diastolic dysfunction; VEDV, left ventricular end-diastolic volume; LVEDVI, left ventricular end-diastolic volume index; LVEF, left ventricular ejection fraction; LVESV, left ventricular end-systolic volume; LVESVI, left ventricular end-systolic volume index; MCR, mineralocorticoid receptor; MR, mitral valve regurgitation; Non-PAF, non-paroxysmal atrial fibrillation (meaning persistent atrial fibrillation or long-standing persistent atrial fibrillation); NYHA, New York Heart Association; PAF, paroxysmal atrial fibrillation; peak TRV, peak tricuspid valve regurgitation velocity; Pre-AAD, oral administration of antiarrhythmic drug before the procedure; Pre-rEF, Pre-ablation reduced ejection fraction (left ventricular ejection fraction <40%); Septal e′ peak velocity, septal early diastolic mitral annular velocity; SGLT-2, sodium glucose cotransporter 2; SHD, structural heart disease.
